# The suspension and nonimplementation of life support interventions in palliative care

**DOI:** 10.62675/2965-2774.20240196Ed-en

**Published:** 2024-08-28

**Authors:** Regis Goulart Rosa, Livia Biason, Lara Patricia Kretzer

**Affiliations:** 1 Hospital Moinhos de Vento Department of Internal Medicine Porto Alegre RS Brazil Department of Internal Medicine, Hospital Moinhos de Vento - Porto Alegre (RS), Brazil.; 2 Hospital Moinhos de Vento Department of Intensive Medicine Porto Alegre RS Brazil Department of Intensive Medicine, Hospital Moinhos de Vento - Porto Alegre (RS), Brazil.; 3 Grupo Baía Sul Intensive Care Units Florianópolis SC Brazil Intensive Care Units, Grupo Baía Sul - Florianópolis (SC), Brazil.

In intensive care units (ICUs), decisions about the suspension and nonimplementation of life support interventions are frequently made and represent some of the greatest challenges faced by health professionals.^(1)^

In this issue of Critical Care Science, the National Academy of Palliative Care (ANCP - *Academia Nacional de Cuidados Paliativos*) expresses its position on the suspension and nonimplementation of life support interventions.^(2)^ The document addresses the ethical complexities involved in decision-making concerning the management of interventions for patients with serious diseases, offering a solid basis for guiding ethical and humane care practices in the context of palliative care in ICUs.

In the document, the ANCP positions itself in the face of the bioethical debate on the differences between withdrawal measures and nonimplementation of life support interventions in the sense that they are morally equivalent, supporting multidisciplinary deliberations in situations in which different perceptions of the moral weight of the two measures are sources of discomfort, conflicts and deliberative delays. On the other hand, the ANCP position emphasizes that both measures are inadequate when they disagree with the values and goals of the patient and/or family, emphasizing the moral importance of the shared decision as a method of defining the goals of care.

The direct discussion of the subject with lucid patients in the ICU who wish to have such discussion should be encouraged so that the opportunity to understand the patients’ values while they have decision-making capacity is not lost, facilitating therapeutic adequacy as the disease progresses. In a society characterized by a plurality of values, it is essential that the concepts of suffering and dysthanasia are adjusted to the patient's perspectives, thus ensuring individuals’ exercise of autonomy.

The document also clarifies the issue of conscientious objection as a measure available to professionals who feel uncomfortable with some deliberation about the suspension or nonimplementation of life support and offers instruction on the conditions and limitations in the use of the measure. Quite significantly, the document also emphasizes that the ethical commitment to the relief of suffering should not be influenced by the decision of whether or not to employ life support interventions.

Importantly, professionals must be aware of the key concepts and basic language of palliative care, as well as communication techniques for the adequate transmission of information to prevent errors in decision-making, as many requests for futile or even potentially inappropriate treatments of patients or family members arise from communication failure. Wrong decisions often result in potentially avoidable suffering for patients, families and health professionals, in addition to wasting resources and contributing to inequity in access to care. Palliative actions should permeate the work of health professionals as a whole, especially within ICUs where all patients have serious life-threatening diseases.

By establishing these concepts, the position paper also explains the difference between futile treatment in the strict sense and potentially inappropriate treatment. The former is always considered a technical and bioethical error and should not be implemented or maintained under any circumstances, not even at the request of the patient and/or family. On the other hand, potentially inappropriate treatments deserve complex deliberation and may become appropriate according to the characteristics and values of the patient and family. Many decisions are made by multidisciplinary teams in ICUs referring to the latter concept; therefore, professionals should give due attention to deliberative skills so that prudent decisions can be made.

Thus, efforts to align technical decisions with the patient's values and goals should guide the care plan. This approach is essential to ensure that care is centered on the person, respecting individuals’ dignity and autonomy and providing relief from suffering in various dimensions: physical, emotional, spiritual and social.^(3)^

The publication of this position is relevant in the current context, marked by the recent publication of the National Policy on Palliative Care,^(4)^ which establishes principles that agree with the ANCP position and emphasizes the importance of early initiation of palliative care together with disease treatment. The practice of palliative care goes far beyond a one-way action aimed at withdrawing or not initiating some measure and includes a range of actions that aim to prevent and alleviate suffering related to serious diseases by finding the most appropriate treatment for patients. For this purpose, deliberative processes that include patients and family members regarding the ends and technical decisions on the most appropriate means are necessary.

For health professionals working in ICUs, where complex and ethical decisions are made daily, the dissemination and implementation of the ANCP guidelines and the National Palliative Care Policy are important guidelines. These professionals often face ethical dilemmas and need to be prepared to make decisions that respect the dignity and wishes of patients. Continuing education, raising awareness about bioethical principles and implementing the guidelines cited here are essential to improve the quality of care provided and ensure that the decisions made are as appropriate as possible. The adoption of these humanized and person-centered practices not only improves the quality of care but also promotes an environment of respect and dignity.

By integrating the position of the ANCP and the National Policy on Palliative Care into clinical practice, we are taking an important step forward in intensive care medicine in Brazil. These documents provide an ethical and practical foundation for health professionals to make well-informed and compassionate decisions, reducing patients’ suffering and respecting their autonomy. The dissemination and implementation of these guidelines should be a priority for everyone involved in the care of critically ill patients, promoting a model of care that recognizes and values life while accepting death as a natural and inevitable process.
