# Supplementary material for: The suspension and nonimplementation of life support interventions in palliative care
Source: Crit Care Sci. 2024 Aug 28;36:e20240196Eden. doi: 10.62675/2965-2774.20240196Ed-en (PMC11463988; doi:10.62675/2965-2774.20240196Ed-en)
Supplement: Translation

# Translation: A suspensão e a não implementação de intervenções de suporte de vida em cuidados paliativos

**DOI:** 10.62675/2965-2774.20240196Ed-pt

**Authors:** Regis Goulart Rosa, Livia Biason, Lara Patricia Kretzer

**Affiliations:** 1 Porto Alegre RS Brasil Departamento de Medicina Interna, Hospital Moinhos de Vento - Porto Alegre (RS), Brasil.; 2 Porto Alegre RS Brasil Departamento de Medicina Intensiva, Hospital Moinhos de Vento - Porto Alegre (RS), Brasil.; 3 Florianópolis SC Brasil Unidades de Terapia Intensiva, Grupo Baía Sul - Florianópolis (SC), Brasil.

Em unidades de terapia intensiva (UTIs), as decisões sobre a suspensão e a não implementação de intervenções de suporte de vida são tomadas com frequência e representam alguns dos maiores desafios enfrentados pelos profissionais de saúde.^(1)^

Nesta edição da *Critical Care Science*, a Academia Nacional de Cuidados Paliativos (ANCP) manifesta seu posicionamento sobre a suspensão e a não implementação de intervenções de suporte de vida.^(2)^ O documento aborda as complexidades éticas envolvidas na tomada de decisões sobre o manejo de intervenções em pacientes com doenças graves, oferecendo uma base sólida para orientação da prática assistencial ética e humanizada no âmbito dos cuidados paliativos em UTIs.

No documento, a ANCP se posiciona diante do debate bioético sobre diferenças entre medidas de retirada e não implementação de intervenções de suporte de vida, no sentido de que são moralmente equivalentes, amparando as deliberações multidisciplinares naquelas situações em que diferentes percepções sobre o peso moral das duas medidas constituem fontes de desconforto, conflitos e atrasos deliberativos. Por outro lado, o posicionamento da ANCP enfatiza que ambas as medidas são inadequadas quando em desacordo com valores e objetivos do paciente e/ou familiares, enfatizando a importância moral da decisão compartilhada como método de definição quanto aos objetivos do cuidado.

A discussão sobre o assunto diretamente com pacientes lúcidos na UTI e que desejam tal discussão deve ser incentivada, para que não se perca a oportunidade de compreender os valores de pacientes enquanto houver capacidade decisória, facilitando a adequação terapêutica mediante à evolução da doença. Em uma sociedade caracterizada pela pluralidade de valores, é essencial que os conceitos de sofrimento e de distanásia sejam ajustados às perspectivas do paciente, assegurando seu exercício de autonomia.

O documento elucida, ainda, a questão da objeção de consciência como medida disponível aos profissionais que se sentem desconfortáveis com alguma deliberação acerca da suspensão ou da não implementação de suporte de vida, ao mesmo tempo que instrui sobre as condições e as limitações no uso da medida. De maneira bastante relevante, o documento ressalta ainda que o compromisso ético pelo alívio do sofrimento não deve ser influenciado pela decisão por empregar ou não intervenções de suporte de vida.

É importante reconhecer que os profissionais tenham claro os conceitos-chave e a linguagem básica dos cuidados paliativos, assim como técnicas de comunicação para adequada transmissão da informação, prevenindo erros na tomada de decisão, visto que muitas solicitações por tratamentos fúteis ou mesmo potencialmente inapropriados por parte dos pacientes ou familiares advêm de falhas de comunicação. Decisões equivocadas frequentemente resultam em sofrimento potencialmente evitável para pacientes, familiares e profissionais de saúde, além de causar desperdício de recursos e contribuir para a inequidade no acesso aos cuidados. Ações paliativas devem permear a atuação dos profissionais de saúde como um todo, mas especialmente dentro das UTIs onde todos os pacientes possuem doenças graves ameaçadoras da vida.

Ao estabelecer esses conceitos, o posicionamento também explicita a diferença entre tratamento fútil em senso estrito e potencialmente inapropriado. O primeiro será sempre considerado um erro tanto técnico quanto bioético e não deve ser implementado ou mantido em hipótese alguma, nem mesmo a pedido do paciente e/ou familiares. Já os tratamentos potencialmente inapropriados merecem uma deliberação complexa e poderão se tornar apropriados de acordo com a biografia e consequentemente os valores do paciente e da família. Grande parte das decisões das equipes multidisciplinares nas UTIs se refere a esse último conceito, e, portanto, a devida atenção dos profissionais deve ser voltada às habilidades deliberativas de maneira que decisões prudentes possam ocorrer.

Assim, alinhar as decisões técnicas com os valores e os objetivos do paciente deve ser o norteador do plano de cuidados. Esse enfoque é fundamental para garantir que o cuidado seja centrado na pessoa, respeitando sua dignidade e autonomia e proporcionando alívio do sofrimento em suas várias dimensões: física, emocional, espiritual e social.^(3)^

A publicação deste posicionamento é relevante no contexto atual, marcado pela recente publicação da Política Nacional de Cuidados Paliativos,^(4)^ que estabelece princípios que vão ao encontro do posicionamento da ANCP e enfatiza a importância de iniciar precocemente os cuidados paliativos com o tratamento da doença. A prática dos cuidados paliativos vai muito além de uma ação unidirecional com intuito de retirar ou não iniciar alguma medida e se trata de uma gama de ações que objetivam prevenir e aliviar o sofrimento relacionado a doenças graves, encontrando o tratamento mais adequado para os pacientes. Para isso, são necessários processos deliberativos que incluam pacientes e familiares quanto aos fins e às decisões técnicas sobre os meios mais apropriados.

Para os profissionais de saúde que atuam em UTIs, onde decisões complexas e éticas são tomadas diariamente, a disseminação e a implementação das diretrizes da ANCP e da Política Nacional de Cuidados Paliativos são norteadores importantes. Esses profissionais frequentemente enfrentam dilemas éticos e precisam estar preparados para tomar decisões que respeitem a dignidade e os desejos dos pacientes. A formação contínua, a sensibilização sobre os princípios bioéticos e a implementação das diretrizes aqui citadas são fundamentais para melhorar a qualidade dos cuidados prestados e garantir que as decisões tomadas sejam as mais adequadas possíveis. A adoção dessas práticas humanizadas e centradas na pessoa não apenas melhora a qualidade do cuidado, mas também promove um ambiente de respeito e dignidade.

Ao integrar o posicionamento da ANCP e a Política Nacional de Cuidados Paliativos na prática clínica, damos um passo importante para a medicina intensiva no Brasil. Esses documentos fornecem um alicerce ético e prático para que os profissionais de saúde possam tomar decisões bem-informadas e compassivas, reduzindo o sofrimento dos pacientes e respeitando sua autonomia. A disseminação e a implementação dessas diretrizes devem ser uma prioridade para todos os envolvidos no cuidado de pacientes críticos, promovendo um modelo de atenção que reconhece e valoriza a vida, ao mesmo tempo que aceita a morte como um processo natural e inevitável.
